# *Lacticaseibacillus rhamnosus* CBT LR5 with skim milk alleviates scopolamine-induced cognitive impairment in mice

**DOI:** 10.3389/fmicb.2025.1672153

**Published:** 2025-10-17

**Authors:** Ho Jung Bae, Song-In Kim, So-Yeon Kim, Ye Eun Cho, Soohyun Sung, Seokhee Lim, Kyohee Cho, Se Jin Park, Sanghyun Lim

**Affiliations:** ^1^School of Interdisciplinary Natural Science with Flexible Major, Glocal Advanced Institute of Science and Technology, Changwon National University, Changwon, Republic of Korea; ^2^R&D Center, Cell Biotech Co., Ltd., Gimpo-si, Republic of Korea; ^3^Agriculture and Life Science Research Institute, Kangwon National University, Chuncheon-si, Republic of Korea; ^4^Department of Food Biotechnology and Environmental Science, Kangwon National University, Chuncheon-si, Republic of Korea

**Keywords:** *Lacticaseibacillus rhamnosus* CBT LR5, probiotics, cognitive functions, gut-brain axis, brain-derived neurotrophic factor

## Abstract

**Introduction:**

Emerging evidence highlights the gut-brain axis as a pivotal pathway linking gastrointestinal health with cognitive function, particularly in neurodegenerative conditions such as Alzheimer’s disease (AD).

**Methods:**

This study investigated the cognitive-enhancing effects of the probiotic strain *Lacticaseibacillus rhamnosus* CBT LR5 (LR5), alone or in combination with skim milk, in a mouse model of scopolamine-induced cognitive impairment. The cognitive functions were evaluated using the novel object recognition test (NOR) and the passive avoidance test (PAT).

**Results:**

The results demonstrated that the oral administration of LR5, especially when combined with skim milk, significantly ameliorated scopolamine-induced cognitive deficits. Mechanistically, treatment with LR5 combined with skim milk restored the diversity and composition of the gut microbiota increased the abundance of beneficial genera, such as *Muribaculaceae* and enhanced intestinal barrier integrity by increasing the expression of tight junction proteins, including claudin-1, occludin, and zonula occludens-1. Additionally, this combination reduced systemic inflammation by lowering serum TNF-*α* and PGE_2_ levels and promoted increased expression of BDNF by activating the CREB-BDNF-TrkB signaling pathway in hippocampal and cortical tissues. Furthermore, correlation analyses revealed significant associations between specific gut bacterial genera, such as *Lacticaseibacillus*, *Turicibacter*, *Cryptobacteroides*, *Ruminococcus*, and *Muribaculaceae*, and cognitive or inflammatory biomarkers.

**Discussion:**

Collectively, these findings suggest that the synergistic effects of *L. rhamnosus* CBT LR5 combined with skim milk may represent an effective dietary intervention for cognitive enhancement, potentially through gut microbiota modulation, improved barrier integrity, reduced inflammation, and enhanced neurotrophic signaling.

## 1 Introduction

The gastrointestinal tract is increasingly recognized as a critical regulator of systemic health, influencing immune responses, metabolism and neurocognitive processes, particularly within the context of neurodegenerative diseases such as Alzheimer’s disease (AD) (Loh et al., 2024; Rob et al., 2025). Central to this regulatory role is the intestinal epithelium, a single-cell layer maintained by specialized tight junction complexes composed of proteins, including claudins, occludin, and zonula occludens-1 (ZO-1) (Ghosh et al., 2021; Chelakkot et al., 2018). These tight junction complexes regulate paracellular permeability, selectively restricting the passage of luminal antigens, the gut microbiota, and toxins into the host circulation. Disruption of tight junction integrity, often termed “leaky gut,” allows bacterial components and proinflammatory cytokines to translocate into systemic circulation, potentially exacerbating systemic inflammation (Barbara et al., 2021; Schoultz and Keita, 2020). In addition, increasing evidence suggests that chronic systemic inflammation resulting from impaired gut barrier function significantly contributes to AD pathology by promoting neuroinflammation and accelerating cognitive decline (Xie et al., 2021; Liu et al., 2020).

*Lacticaseibacillus rhamnosus* has garnered considerable attention because of its beneficial roles in modulating the gut microbiota, reducing systemic inflammation, and producing neuroactive compounds (Tette et al., 2022), in part through enhancing tight junction protein expression and thus reinforcing intestinal barrier integrity (Zheng et al., 2022; Bhat et al., 2020). Recent evidence indicates that supplementation with *L. rhamnosus* may improve mental health outcomes, including reducing anxiety and enhancing memory by promoting neurogenesis and synaptic plasticity, both of which are fundamental for learning and memory (Feng et al., 2025; Isik et al., 2025; Guimaraes et al., 2025). Notably, Xiao-Hang et al. (2024) reported significant cognitive improvements following the administration of multistrain probiotics containing *Bifidobacterium lactis* and *L. rhamnosus* in SAMP8 and SAMP1 mice, which are animal models of AD. Clinical studies have further supported these findings, showing beneficial effects of *L. rhamnosus* supplementation on cognitive function in patients diagnosed with AD or mild cognitive impairment (Aljumaah et al., 2022; Akhgarjand et al., 2022). Additionally, animal studies have demonstrated that cognitive enhancement by *L. rhamnosus* GG in models of noise-induced cognitive deficits and sepsis can be attributed primarily to the modulation of the systemic inflammatory response (Wang et al., 2024; Li X. et al., 2023). These findings highlight the therapeutic potential of *L. rhamnosus* for cognitive impairments associated with AD.

On the other hand, milk is rich in nutrients and bioactive compounds capable of positively influencing the gut microbiome (Gallo et al., 2024; Zheng et al., 2024). Milk-derived proteins, lipids, and natural prebiotics, such as lactose and oligosaccharides, selectively promote the growth of beneficial gut microbiota (Walsh et al., 2020). Additionally, immunomodulatory components including lactoferrin help maintain a balanced gut microbiome by regulating microbial composition and enhancing host immunity (Demir et al., 2025). These characteristics underscore the integral role of milk in shaping a healthy intestinal environment. Therefore, we hypothesized that the combination of *L. rhamnosus* and milk would exert synergistic effects, enhancing cognitive function by beneficially altering the gut microbiota composition, reinforcing intestinal barrier integrity, attenuating systemic inflammation and modulating critical determinants of brain health. However, the mechanisms underlying these beneficial effects of *L. rhamnosus* combined with milk in animal models of AD remain unexplored.

In this study, we investigated whether supplementation with *L. rhamnosus* in combination with milk could mitigate the cognitive impairment induced by scopolamine, a commonly utilized model reflecting the cognitive deficits and pathophysiological features observed in patients with AD. Behavioral assessments, including novel object recognition (NOR) and passive avoidance tests, were conducted to evaluate cognitive outcomes. Additionally, molecular analyses employing Western blot, polymerase chain reaction, and enzyme-linked immunosorbent assays (ELISAs) were performed to elucidate the underlying mechanisms through which the combination of *L. rhamnosus* and milk influences cognitive function.

## 2 Materials and methods

### 2.1 Animals

Male C57BL/6 mice (18–20 g) were obtained from Orient Co., Ltd., a subsidiary of Charles River Lab. (Seongnam-si, Gyeonggi-do, Korea). The animals were housed in groups of five per cage under controlled conditions (temperature: 23 ± 1 °C; humidity: 60 ± 10%; 12 h light/dark cycle with lights on from 07:00 to 19:00) at the Animal Care Unit of Kangwon National University. Food and water were provided *ad libitum*. The mice were acclimatized to laboratory conditions for 1 week prior to experimentation. All experimental procedures complied with the Animal Care and Use Guidelines established by Kangwon National University. The study protocol received approval from the Institutional Animal Care and Use Committee of Kangwon National University (Approved No. KW-220830-1).

### 2.2 Materials

All chemical reagents and materials used in this study were of analytical grade or higher. Scopolamine and donepezil were purchased from Sigma-Aldrich Co. (St. Louis, MO, United States). The probiotic strain *Lacticaseibacillus rhamnosus* CBT LR5 (LR5, KCTC 12202BP), which was isolated from human fecal samples in Korea, was provided by Cell Biotech (Gimpo-si, Gyeonggi-do, Korea) and is listed as Generally Recognized As Safe (GRAS) by the U.S. Food and Drug Administration (FDA). Skim milk (non-fat dry milk) including lactose contents 40–60% was used in this study. The antibodies used included anti-phospho-CREB (#9198), anti-CREB (#9197), anti-GAPDH (#2118), and anti-rabbit IgG antibodies purchased from Cell Signaling Technology (Danvers, MA, United States) and anti-BDNF (#ab108319) antibody purchased from Abcam (Cambridge, MA, United States).

### 2.3 Treatment and the experimental schedule

After a one-week acclimatization period, the mice were weighed and evenly assigned to six experimental groups to ensure comparable average body weights. The groups included vehicle-treated control (Con), scopolamine-treated (Sco), scopolamine-treated with skim milk (SK), scopolamine-treated with *L. rhamnosus* CBT LR5 (LR5), scopolamine-treated with combination of *L. rhamnosus* CBT LR5 and skim milk (LR5 + SK) and scopolamine-treated with donepezil (DNZ).

**Figure 1:**
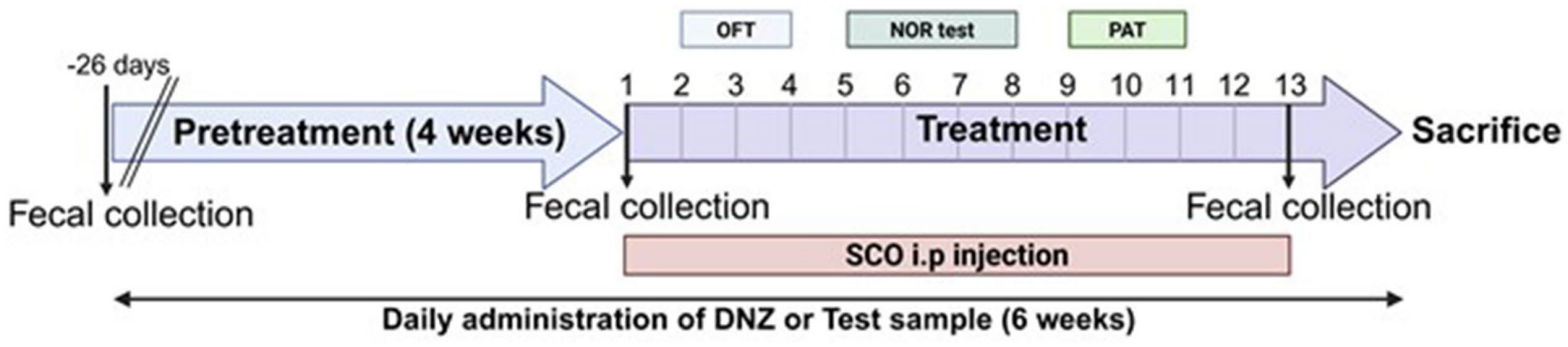
Scheme of the experiment. The experimental schedule employed in the present study is illustrated.

Treatment with the test substances was initiated and continued for 4 weeks. To induce memory impairments, scopolamine (1 mg/kg) was intraperitoneally (i.p.) treated to the mice. The mice in the LR5 and LR5 + SK groups received daily oral administration of *L. rhamnosus* CBT LR5 at a dose of 1 × 10^9^ CFU/day. The DNZ group, which served as a positive control, received donepezil (5 mg/kg/day, p.o.). The vehicle-treated control and Sco groups were administered 0.9% saline solution. Throughout this treatment period, body weights were monitored every 5 days to detect any adverse reactions or toxicity (Supplementary Figure S1). Following the 4-week treatment regimen, cognitive impairment was induced by scopolamine administration in all groups except the vehicle-treated control group. Subsequently, behavioral tests were conducted sequentially over a two-week period, progressing from the least to the most stressful conditions as follows, the open field test, novel object recognition test, and passive avoidance test (Figure 1).

After the behavioral assessments, intestinal and brain tissues, as well as blood samples, were collected from each animal for biochemical and molecular analyses. Fecal samples were collected at three time points, including prior to treatment initiation, following the treatment period but before scopolamine administration, and immediately before tissue collection.

### 2.4 Behavioral tests

#### 2.4.1 Novel object recognition test

The NOR test was performed to evaluate recognition memory, following previously established protocols with minor modifications (Bae et al., 2023). The apparatus consisted of a black polyvinyl plastic square box (30 × 30 × 30 cm) with an open top. The test comprises three phases, habituation, training and probe trials. Each mouse was first habituated to the empty box for 10 min a day. During the training session, the mice were allowed to explore the empty box for 5 min before being introduced to two identical objects placed symmetrically within the apparatus for an additional 5 min. After a 24 h retention interval, the probe trial was conducted by replacing one familiar object with a novel object. Each mouse was then reintroduced into the apparatus and allowed to explore freely for 5 min. All the objects used were similar in size, texture and color to prevent bias. The apparatus and objects were cleaned thoroughly with 70% ethanol between trials to eliminate olfactory cues. Behavioral activity was recorded using an Etho-Vision video-tracking system (Noldus, Wageningen, Netherlands) and analyzed by a person who was blinded to the treatment. The exploration times spent on the novel object (T_novel_) and the familiar object (T_familiar_) were recorded. Recognition memory was assessed using the discrimination ratio, which was calculated as follows:


$$\text{Discrimination ratio},(T_{\text{novel}}-T_{\text{familiar}})/(T_{\text{novel}}+T_{\text{familiar}})\times 100\;(\%)$$

Object preference ratio, the percentage of exploration time directed toward either the novel or familiar object, calculated as


$$T_{\text{object}}/(T_{\text{novel}}+T_{\text{familiar}})\times 100\;(\%)$$

Total exploration time, the sum of exploration times for both objects (T_novel_ + T_familiar_).

#### 2.4.2 Passive avoidance test

The passive avoidance test (PAT) was employed to assess long-term memory retention and was conducted over two consecutive days, including acquisition and retention trials (Bae et al., 2020). The apparatus consisted of two connected chambers (each 20 × 20 × 20 cm) separated by a sliding door (5 × 5 cm). One chamber was brightly illuminated (light chamber) with a 50 W white LED bulb, and the entire adjacent chamber remained dark (dark chamber). The floors of both chambers were composed of stainless-steel bars (2 mm diameter) spaced 1 cm apart, which delivered a mild foot shock.

During the acquisition trial, each mouse was placed individually into the illuminated chamber, and after 10 s, the connecting door was opened. When the mouse completely entered the dark chamber, the door closed automatically, and the mouse received a single mild foot shock (0.5 mA, 3 s). If a mouse failed to enter the dark chamber within 60 s, it was gently guided into the dark chamber, and the latency was recorded as 60 s. Thereafter, the retention trial was conducted 24 h later, where each mouse was placed again into the illuminated chamber. The latency to enter the dark chamber was measured, similar to the acquisition trial. However, during the retention trial, no foot shock was delivered. Mice that did not enter the dark chamber within the maximum latency period (300 s) were recorded, with a latency of 300 s.

### 2.5 16S rRNA extraction and analysis

Fecal samples were collected from the mice to analyze microbiota composition changes induced by scopolamine or LR5 treatment via 16S rRNA sequencing, as previously described (Jung et al., 2025). Genomic DNA was extracted using a SPINeasy DNA Pro Kit for Soil (MP Biochemicals, Santa Ana, CA, United States) according to the manufacturer’s protocol. Sequencing libraries targeting the V4–V5 hypervariable region of the bacterial 16S rRNA gene were prepared following the Illumina 16S metagenomic sequencing library preparation guidelines. Polymerase chain reaction (PCR) amplification was performed with forward (5′-CCA GCMGCC GCG GTA ATW C-3′) and reverse (5′-CC GTC AAT TYY TTT RAG TTT-3′) primers targeting the V4–V5 region. A Nextera XT v2 Index Kit (Illumina, San Diego, CA, United States) was used for indexing PCR. Next-generation sequencing (NGS) was conducted using the Illumina MiSeq platform (Illumina, San Diego, CA, United States) with paired-end reads (2 × 250 bp) and a MiSeq Reagent Kit V2. The sequencing data were processed and analyzed using QIIME2 software (version 2024.5, viewed on May 3, 2024), and sequence quality filtering was performed with the DADA2 plugin.

### 2.6 Enzyme-linked immunosorbent assay

The levels of inflammatory mediators and neurotrophic biomarkers in the serum were quantified by ELISA following the manufacturer’s instructions. The concentration of tumor necrosis factor-alpha (TNF-*α*, Cat. NO_MTA00B-1, R&D systems, Minneapolis, MN, United States), prostaglandin E_2_ (PGE_2_, Cat. NO_KGE004B, R&D systems, Minneapolis, MN, United States), brain-derived neurotrophic factor (BDNF, Cat. NO_DBNT00, R&D systems, Minneapolis, MN, United States), and acetylcholinesterase (AChE, Cat. NO _BM-ACH-100, BIOMAX, Korea) were measured in the serum samples according to the manufacturers’ instructions. Optical densities were measured using a microplate reader (SpectraMax iMark^™^, Molecular Devices, CA, United States). The concentrations of each biomarker were determined according to standard curves provided by the manufacturers.

### 2.7 Real-time quantitative PCR analysis

Total RNA from mouse brain and ileal tissues were isolated using TRIzol reagent (Invitrogen, Carlsbad, CA, United States) according to the manufacturer’s protocol. The purity and concentration of the extracted RNA were quantified using a SpectraMax^®^ QuickDrop^™^ UV–Vis Spectrophotometer (Molecular Devices, San Jose, CA, United States). Complementary DNA (cDNA) synthesis was performed using a PrimeScript^™^ RT reagent Kit (Takara Bio Inc., Shiga, Japan) following the manufacturer’s instructions. Real-time quantitative PCR analysis (RT-qPCR) was conducted on a CFX Opus 96 Real-Time PCR System (Bio-Rad Laboratories, Hercules, CA, United States) under the following cycling conditions: initial denaturation at 95 °C for 10 min, followed by 40 cycles of denaturation at 95 °C for 5 s, annealing at 58 °C for 25 s, and extension at 72 °C for 3 s. Each reaction mixture was prepared with SYBR Green-containing master mix (Takara Bio Inc.), primers specific for the target genes (Table 1), and a cDNA template. Relative gene expression levels were normalized to those of GAPDH, a housekeeping gene, and analyzed using the comparative 2^−ΔΔCT^ method (Turabelidze et al., 2010).

**Table 1:** RT-qPCR primers.

Gene	Primer sequences	Reference
*ZO-1*	F: GTTGGTACGGTGCCCTGAAAGA	Dong et al. (2022)
	R: GCTGACAGGTAGGACAGACGAT	
*Occludin*	F: TGGCAAGCGATCATACCCAGAG	
	R: CTGCCTGAAGTCATCCACACTC	
*Claudin-1*	F: GGACTGTGGATGTCCTGCGTTT	Pang et al. (2022)
	R: GCCAATTACCATCAAGGCTCGG	
*Gapdh*	F: CATCACTGCCACCCAGAAGACTG	Kurland et al. (2016)
	R: ATGCCAGTGAGCTTCCCGTTCAG	
*L. rhamnosus*	F: CTAGCGGGTGCGACTTTGTT	Ansari et al. (2023)
	R: GCGATGCGAATTTCTATTAT	

### 2.8 Western blotting assay

Brain tissue samples were homogenized in protein extraction buffer consisting of PRO-PREP^™^ Protein Extraction Solution (iNtRON Biotechnology, Seongnam, Korea) supplemented with protease and phosphatase inhibitors, according to the manufacturer’s guidelines and previous studies (Bae et al., 2023; Bae et al., 2020). Protein concentrations were quantified by the Bradford assay. Subsequently, 30 μg of total protein from each sample was separated via sodium dodecyl-sulfate polyacrylamide gel electrophoresis (SDS-PAGE) in 10% acrylamide gels under reducing conditions and transferred to polyvinylidene difluoride (PVDF) membranes. The membranes were blocked for 1 h at room temperature with 5% skim milk in Tween 20/Tris-buffered saline (TTBS) and then incubated overnight at 4 °C with primary antibodies (1:1,000 dilution). After being washed three times for 10 min each with TTBS, the membranes were incubated for 2 h at room temperature with appropriate horseradish peroxidase-conjugated secondary antibodies (1:2,500 dilution). The protein bands were visualized using Clarity^™^ Western ECL Substrate (Bio-Rad Laboratories, CA, United States) and quantified by densitometric analysis using a ChemiDoc^™^ Touch Imaging System (Bio-Rad Laboratories, CA, United States).

### 2.9 Statistics

All the data are presented as the means ± standard error of the mean (S.E.M.). Statistical analyses were performed using GraphPad Prism 8.0 (GraphPad Software Inc., San Diego, CA, United States). The behavioral data were analyzed using one-way analysis of variance (ANOVA), followed by post-hoc analysis with Turkey’s multiple comparisons test. Molecular data obtained from Western blot analysis, qPCR, and ELISA were analyzed by one-way ANOVA followed by Dunnett’s T3 multiple comparisons test. Differences with a *p*-value of less than 0.05 (*p* < 0.05) were considered statistically significant.

## 3 Results

### 3.1 LR5 combined with milk supplementation attenuated object recognition memory in scopolamine-induced cognitive deficit mice

**Figure 2:**
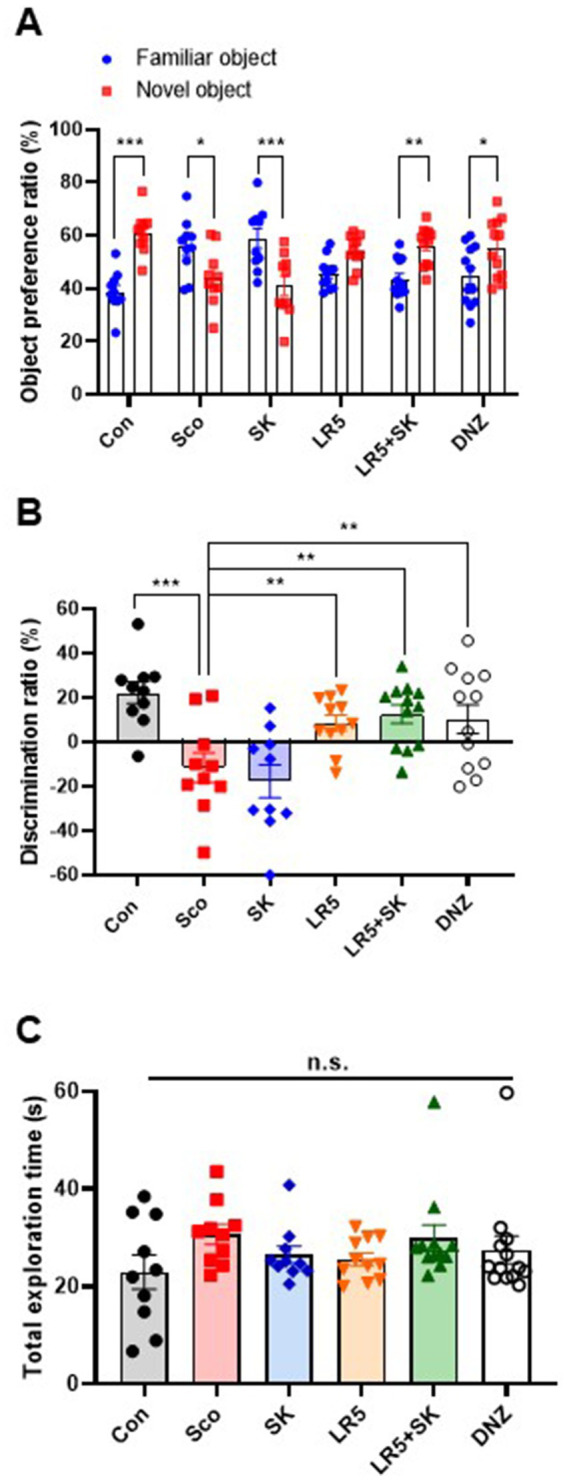
Effects of *L. rhamnosus* CBT LR5 combined with skim milk on the impaired novel object recognition memory induced by scopolamine in the novel object recognition test. The preference ratios of novel and familiar objects **(A)**, discrimination ratios **(B)**, and total exploration times **(C)** are presented. Statistical analysis was performed using one-way ANOVA followed by Turkey’s multiple comparisons test. The data represent the means ± S.E.M. (*n* = 9–10/group) (^*^p < 0.05, ^**^p < 0.01, ^***^p < 0.001; A: versus the other groups). Con, control; Sco, scopolamine; SK, skim milk; LR5, *L. rhamnosus* CBT LR5; DNZ, donepezil.

To investigate the potential beneficial effects of chronic supplementation with LR5 or LR5 + SK on scopolamine-induced cognitive impairment, the NOR test was performed. Statistical analyses revealed significant differences among the experimental groups in terms of the object preference ratio (two-way ANOVA, treatment, *F*_5,118_ = 0, *p* > 0.05; object, *F*_1,118_ = 6.242, *p* < 0.05; interaction _treatment × object_, *F*_5,118_ = 14.01, *p* < 0.0001, Figure 2A) and discrimination ratio (one-way ANOVA, *F*_5,59_ = 7.007, *p* < 0.001, Figure 2B). Specifically, treatment with LR5 or LR5 + SK significantly increased the discrimination ratio compared with that of the scopolamine-treated group (*p* < 0.01), effectively reversing scopolamine-induced deficits in the discrimination ratio. These effects were comparable to those observed in the donepezil-treated group. Additionally, no significant differences in the total exploration time among the groups were noted (Figure 2C), which is consistent with the results obtained from open filed test (Supplementary Figure S2), indicating that locomotor activity did not confound cognitive performance outcomes. Collectively, these results suggest that supplementation with LR5, particularly in combination with skim milk, enhances recognition memory in mice subjected to cholinergic deficits induced by scopolamine.

### 3.2 LR5 combined with milk mitigated contextual long-term memory impairment in scopolamine-induced cognitive deficit mice

**Figure 3:**
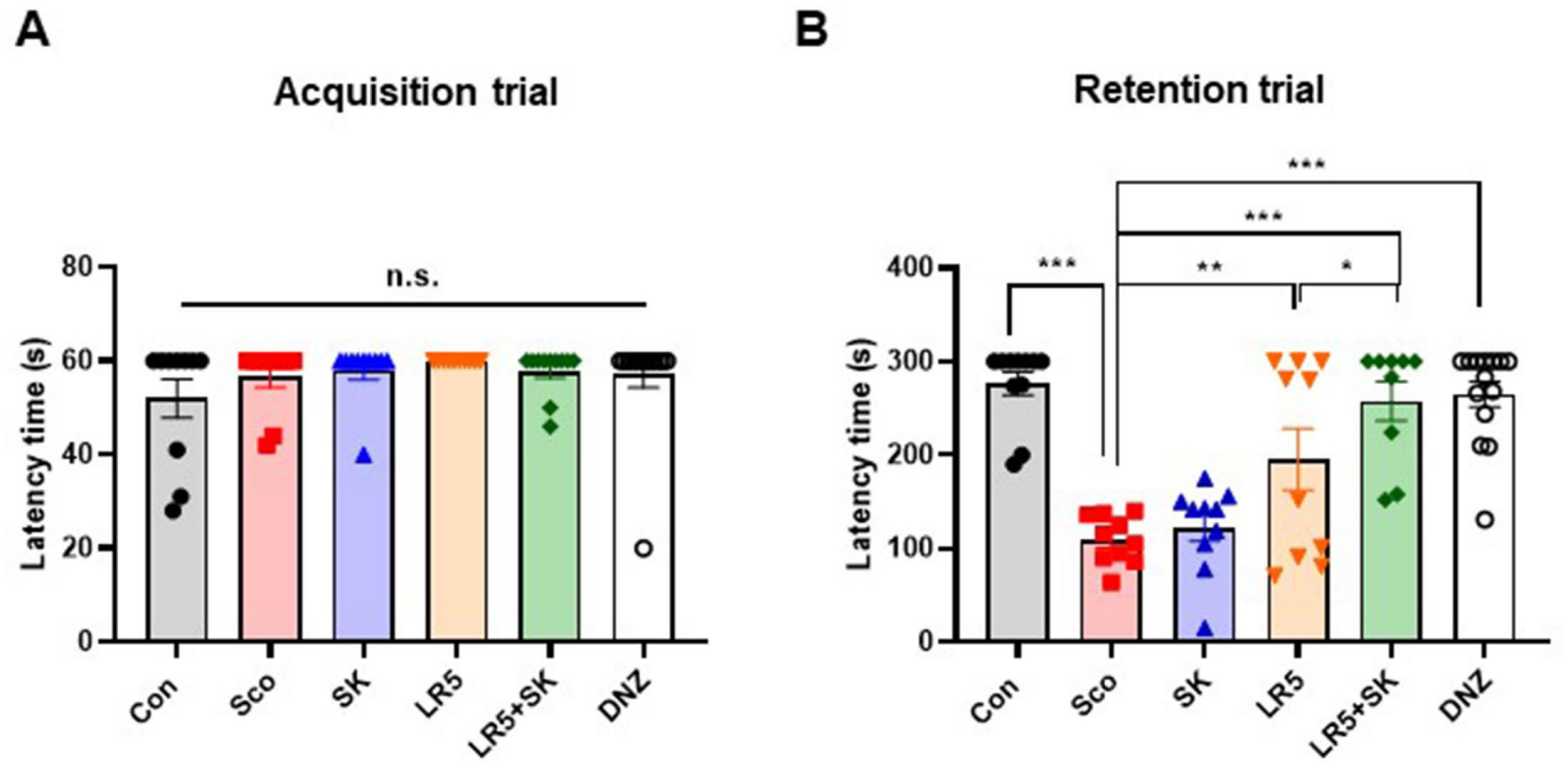
Effects of *L. rhamnosus* CBT LR5 combined with skim milk on the impaired contextual long-term memory induced by scopolamine in the passive avoidance test. The latencies of the acquisition trial **(A)** and retention trial **(B)** are presented. Statistical analysis was performed using one-way ANOVA followed by Turkey’s multiple comparisons test. The data represent the means ± S.E.M. (*n* = 9–10/group) (^*^p < 0.05, ^**^p < 0.01, ^***^p < 0.001; A: versus the other groups). Con, control; Sco, scopolamine; SK, skim milk; LR5, *L. rhamnosus* CBT LR5; DNZ, donepezil.

The PAT was conducted to evaluate the effects of LR5 or LR5 + SK on contextual long-term memory. Significant differences among the experimental groups were detected during the retention trial (one-way ANOVA, *F*_5,59_ = 4.914, *p* < 0.0001; Figure 3B), whereas no significant differences were detected during the acquisition trial (one-way ANOVA, *F*_5,59_ = 1.024, *p* > 0.05; Figure 3A). Compared with vehicle-treated control mice, scopolamine-treated mice presented significantly shorter latency times, which is indicative of impaired memory retention. Compared with scopolamine alone, treatment with LR5 and LR5 + SK significantly prolonged the latency to enter the dark chamber, suggesting improved memory retention. Notably, the latency in the LR5 + SK group was greater than that in the LR5-only group, suggesting a potential synergistic effect between LR5 and skim milk. Collectively, these results indicate that LR5 supplementation, particularly when combined with skim milk, effectively ameliorates scopolamine-induced contextual long-term memory deficits.

### 3.3 Quantitative analysis of *Lacticaseibacillus* abundance in the gut microbiota

To investigate whether oral administration of LR5 colonized the gut, microbiome analysis was performed, specifically to quantify the relative abundance of the genus *Lacticaseibacillus* in the fecal samples (Supplementary Figure S3). At baseline (0 weeks), *Lacticaseibacillus* was undetectable. However, it was prominently detected following 4 weeks of LR5 administration. Notably, after an additional 2 weeks of scopolamine treatment (6 weeks), which induced gut microbiota dysbiosis, the relative abundance of *Lacticaseibacillus* decreased. Complementary qPCR analysis confirmed a significant increase in the abundance of *Lacticaseibacillus* in LR5 after treatment, with a peak observed at week 4, followed by a decrease after scopolamine administration at week 6 (Table 2). These findings suggest that the colonization and sustained abundance of LR5 in the gut may be associated with cognitive enhancement in mice subjected to scopolamine-induced cognitive impairment.

**Table 2:** LR5 level in fecal samples.

Group	LR5 (log CFU: means ± SD)		
	0 day	4 weeks	6 weeks
Con	1.90 ± 0.37	1.75 ± 0.46	2.42 ± 0.52
Sco	2.06 ± 0.52	2.36 ± 0.32	2.17 ± 0.54
SK	2.02 ± 0.66	2.29 ± 0.51	2.31 ± 0.31
LR5	1.98 ± 0.49	**6.46 ± 0.77** ^***,###^	**4.16 ± 0.55** ^***,###^
LR5 + SK	1.92 ± 0.37	**5.99 ± 0.96** ^***,###^	**4.37 ± 0.56** ^***,###^
DNZ	1.54 ± 0.66	2.14 ± 0.33	1.96 ± 0.72

^*^Represents comparison to the 0 day in same group (^***^p < 0.001). ^#^Represents comparison with Sco group in same time (^###^*p* < 0.001). The data represent the means ± S.E.M. (*n* = 10 per group). Con, control; Sco, scopolamine; SK, skim milk; LR5, *L. rhamnosus* CBT LR5; DNZ, donepezil. The bold valuses provided the significant differences between the control gorups.

### 3.4 LR5 ameliorated scopolamine-induced gut dysbiosis in mice

**Figure 4:**
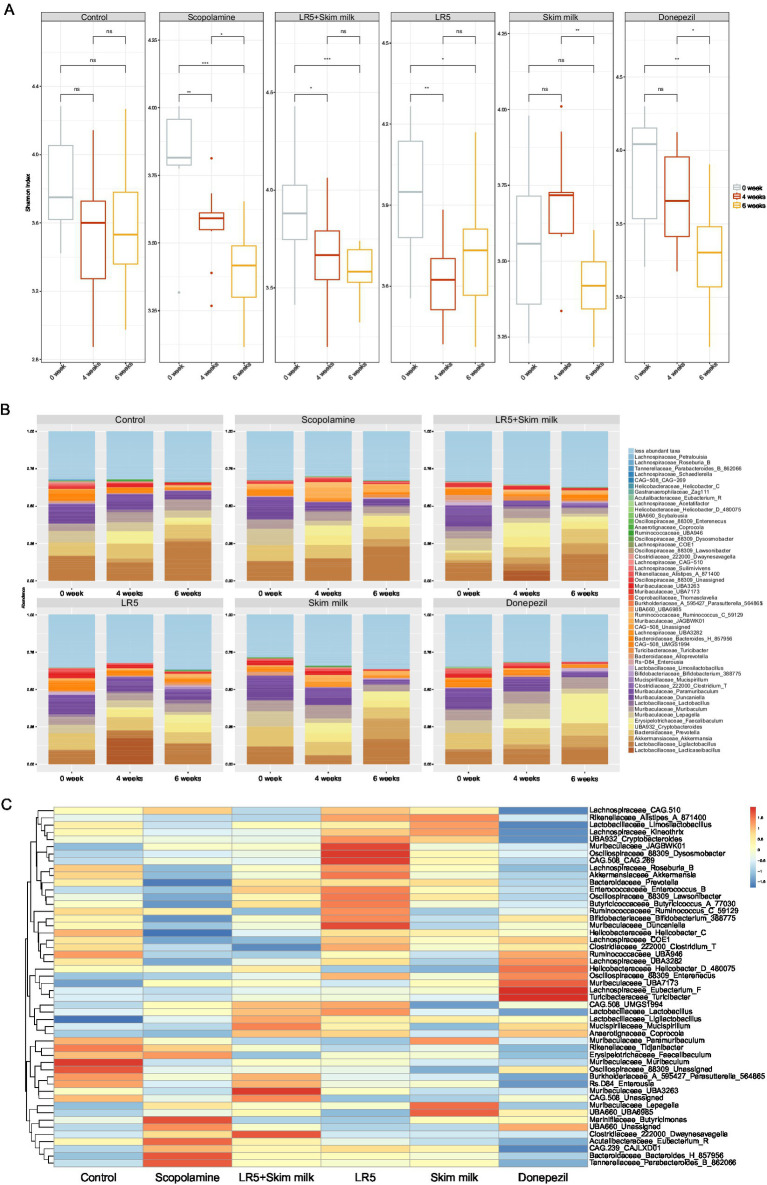
*Lacticaseibacillus rhamnosus* combined with skim milk alleviated scopolamine-induced disturbances in gut permeability and inflammation in mice. The mRNA expression levels of tight junction proteins, including claudin-1 **(A)**, occludin **(B)**, and ZO-1 **(C)**, in ileum tissues are presented. The levels of inflammatory biomarkers, such as PGE_2_ **(D)**, TNF-*α* **(E)**, and BDNF **(F)**, in the serum are presented. The mRNA expression levels of tight junction proteins, including claudin-1 **(G)**, occludin **(H)**, and ZO-1 **(I)**, in hippocampal tissues are presented. Statistical analysis was performed using one-way ANOVA followed by Dunnett’s T3 multiple comparisons test. The data represent the means ± S.E.M. (*n* = 4–5/group) (^*^p < 0.05, ^**^p < 0.01, ^***^p < 0.001; A: versus the other groups). Con, control; Sco, scopolamine; SK, skim milk; LR5, *L. rhamnosus* CBT LR5; DNZ, donepezil.

**Figure 5:**
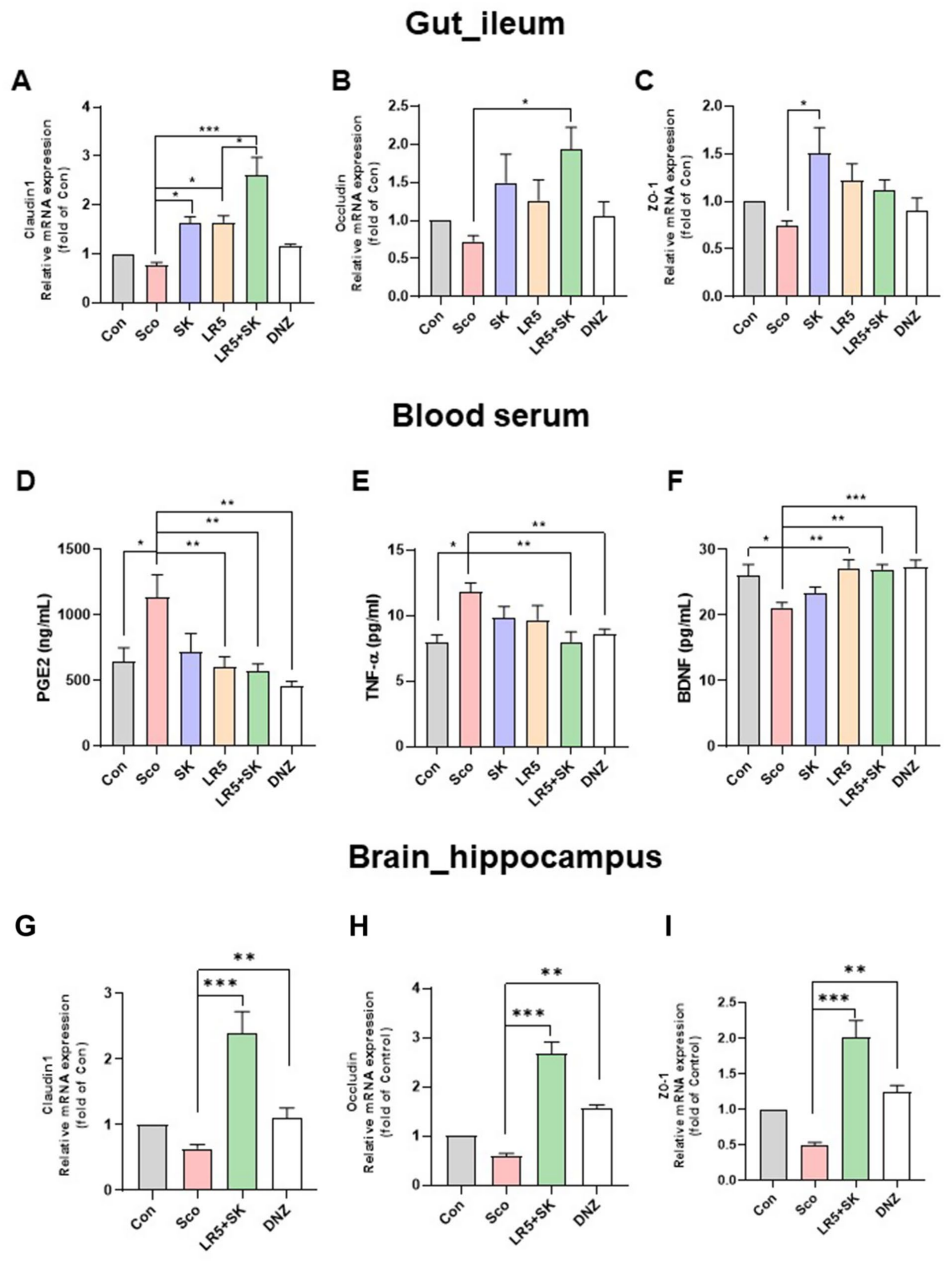
*Lacticaseibacillus rhamnosus* ameliorated scopolamine-induced gut dysbiosis in mice. The Shannon alpha diversity **(A)** and taxonomic profile heatmaps at the genus level **(B)** are presented. Statistical analysis was performed using one-way ANOVA followed by Dunnett’s T3 multiple comparisons test. The data represent the means ± S.E.M. (*n* = 9–10/group) (^*^p < 0.05, ^**^p < 0.01, ^***^p < 0.001; A: versus the other groups). Con, control; Sco, scopolamine; SK, skim milk; LR5, *L. rhamnosus* CBT LR5; DNZ, donepezil.

To elucidate whether the cognitive-enhancing effects of LR5 supplementation were linked to gut microbiota modulation, microbiome analyses via 16S rRNA sequencing were conducted after 4 weeks of probiotic supplementation and a subsequent 2 weeks of scopolamine-induced dysbiosis. The alpha-diversity, assessed by the Shannon diversity index, was significantly lower in the scopolamine-treated group than in the vehicle-treated control group (*p* < 0.01, Figure 4A). However, the administration of LR5 and LR5 + SK significantly attenuated this reduction, effectively restoring microbial diversity (Figure 5A).

Taxonomic profiling at the genus level revealed significant microbiota alterations induced by scopolamine. Specifically, the relative abundances of beneficial genera such as *Muribaculaceae_UBA3263* and *Clostridiaceae_222000_Dwaynesavagella* were notably greater in the groups receiving LR5 + SK than in the scopolamine-treated groups. In contrast, administration of scopolamine only markedly reduced the relative abundance of genera including *Muribaculaceae_JAGBWK01, Oscillospiraceae_88309_Dysosmobacter, CAG.508_CAG.269, and Muribaculaceae_Duncaniella*, all of which tended toward recovery following treatment with LR5. Furthermore, genera such as *Marinifilaceae_Butyricimonas*, *UBA660_Unassigned*, *Clostridiaceae_222000_Dwaynesavagella*, *Acutalibacteraceae_Eubacterium_R*, *CAG.239_CAJLXD01*, *Bacteroidaceae_Bacteroides_H_857956*, and *Tannerellaceae_Parabacteroides_B_862066* exhibited elevated abundances exclusively in the scopolamine-treated groups without probiotic intervention (Figures 5B,C). Taken together, these data strongly suggest that LR5 supplementation mitigates scopolamine-induced gut dysbiosis, thereby potentially contributing to the observed cognitive improvements via the modulation of gut microbiota-mediated mechanisms underlying cognitive improvements.

### 3.5 LR5 in combination with milk alleviated scopolamine-induced disturbances in gut permeability and inflammation in mice

Previous studies have shown that scopolamine-induced gut microbiome dysbiosis disrupts intestinal barrier function and blood–brain barrier (BBB) integrity, thereby exacerbating cognitive impairment via systemic inflammation (Zhang L. et al., 2024; Ni et al., 2024). To explore whether the administration of LR5 or LR5 + SK mitigates these disruptions, we measured the expression levels of tight junction proteins, including claudin-1, occludin, and ZO-1, in ileal and hippocampal tissues (Figures 4A–C). In ileal tissue, qPCR analysis revealed significant group differences in the expression levels of claudin-1 (*F*_5,24_ = 15.75, *p* < 0.001, Figure 4A), occludin (*F*_5,24_ = 3.275, *p* < 0.05, Figure 4B) and ZO-1 (*F*_5,24_ = 3.222, *p* < 0.05, Figure 4C). Scopolamine administration significantly reduced claudin-1 expression, whereas treatment with LR5 or LR5 + SK significantly reversed this reduction. Notably, occludin expression was significantly elevated only with LR5 + SK administration, suggesting potential synergistic protective effects on intestinal permeability.

To further investigate the systemic inflammation potentially induced by the disruption of gut permeability, the serum levels of the inflammatory biomarkers PGE_2_, and TNF-*α*, as well as the peripheral levels of BDNF, were quantified by ELISA (Figures 4D–F). Significant differences among treatment groups were observed for PGE_2_ (one-way ANOVA, *F*_5,48_ = 4.029, *p* < 0.01, Figure 4D), TNF-α (one-way ANOVA, *F*_5,34_ = 3.841, *p* < 0.01, Figure 4E), and BDNF (one-way ANOVA, *F*_5,48_ = 5.349, *p* < 0.001, Figure 4F). Scopolamine treatment significantly elevated the serum PGE_2_ and TNF-α levels, while the administration of LR5 or LR5 + SK effectively attenuated these inflammatory responses, with the combined treatment resulting in a greater reduction in TNF-α. Additionally, scopolamine significantly decreased serum BDNF levels, which were normalized by the LR5 and LR5 + SK treatments.

Furthermore, BBB integrity was assessed by RT-qPCR in hippocampal tissue for tight junction-related gene expression (Figures 4G–I). One-way ANOVA revealed significant differences in the hippocampal expression of claudin-1 (*F*_3,24_ = 17.29, *p* < 0.001, Figure 4G), occludin (*F*_3,24_ = 3.589, *p* < 0.01, Figure 4H) and ZO-1 (*F*_3,24_ = 1.218, *p* < 0.01, Figure 4I). Compared with scopolamine, the administration of LR5 + SK significantly elevated the expression levels of all tight junction proteins, indicating enhanced BBB integrity.

Collectively, these results demonstrate that LR5 combined with skim milk exerts synergistic protective effects on gut and BBB integrity, along with attenuating systemic inflammation, potentially mediating the cognitive improvement observed in individuals with scopolamine-induced cognitive impairment.

### 3.6 LR5 combined with skim milk alleviated the scopolamine-induced reduction in BDNF and related signaling molecules in the brain

**Figure 6:**
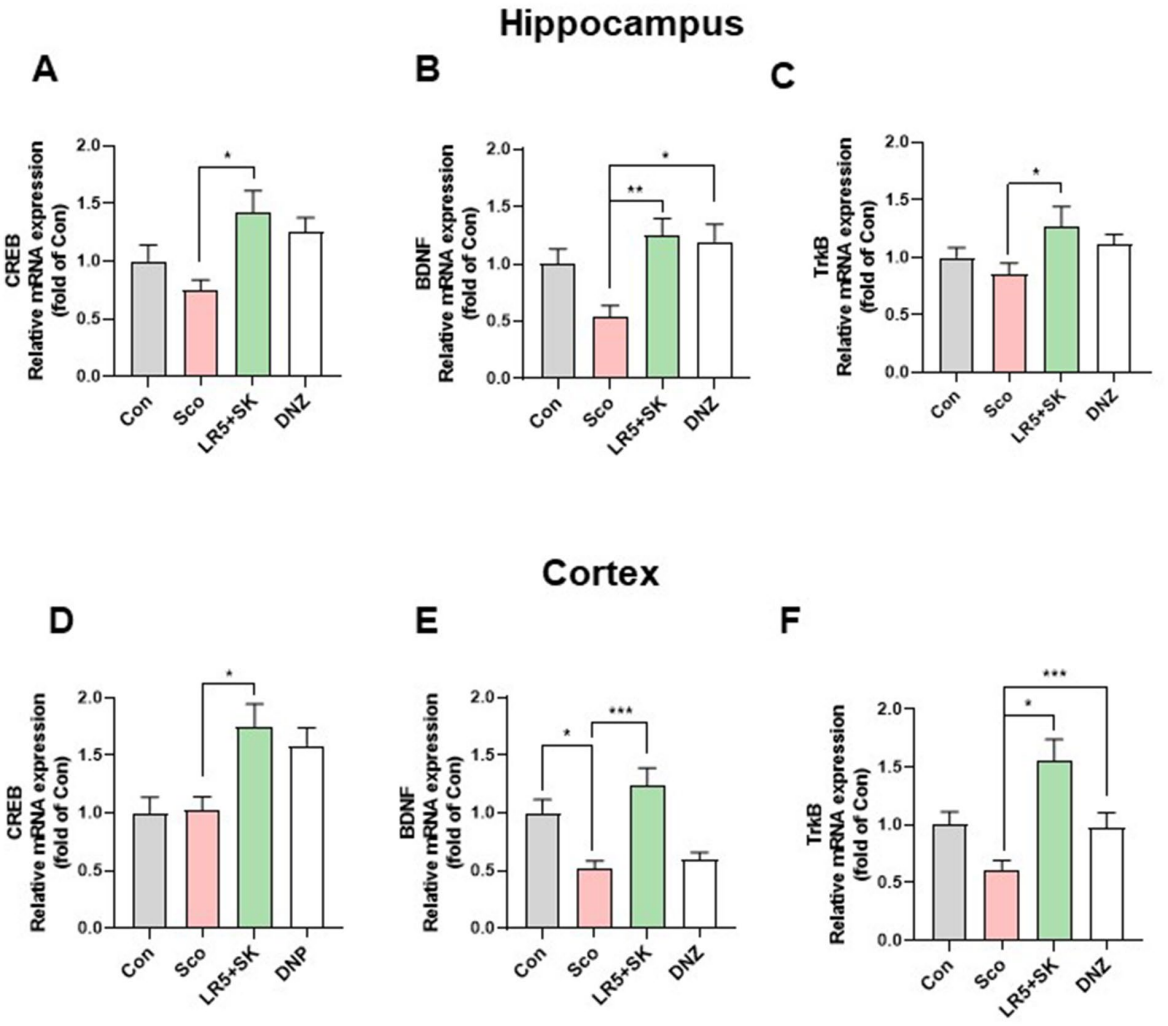
Effects of *L. rhamnosus* combined with skim milk on BDNF, CREB, and TrkB mRNA expression levels in hippocampal and cortical tissues. The mRNA expression levels of CREB **(A)**, BDNF **(B)**, and TrkB **(C)** in hippocampal tissues are presented. The mRNA expression levels of CREB **(D)**, BDNF **(E)**, and TrkB **(F)** in cortical tissues are presented. Statistical analysis was performed using one-way ANOVA followed by Dunnett’s T3 multiple comparisons test. The data represent the means ± S.E.M. (*n* = 4–5/group) (^*^p < 0.05, ^**^p < 0.01, ^***^p < 0.001; A: versus the other groups). Con, control; Sco, scopolamine; SK, skim milk; LR5, *L. rhamnosus* CBT LR5; DNZ, donepezil.

**Figure 7:**
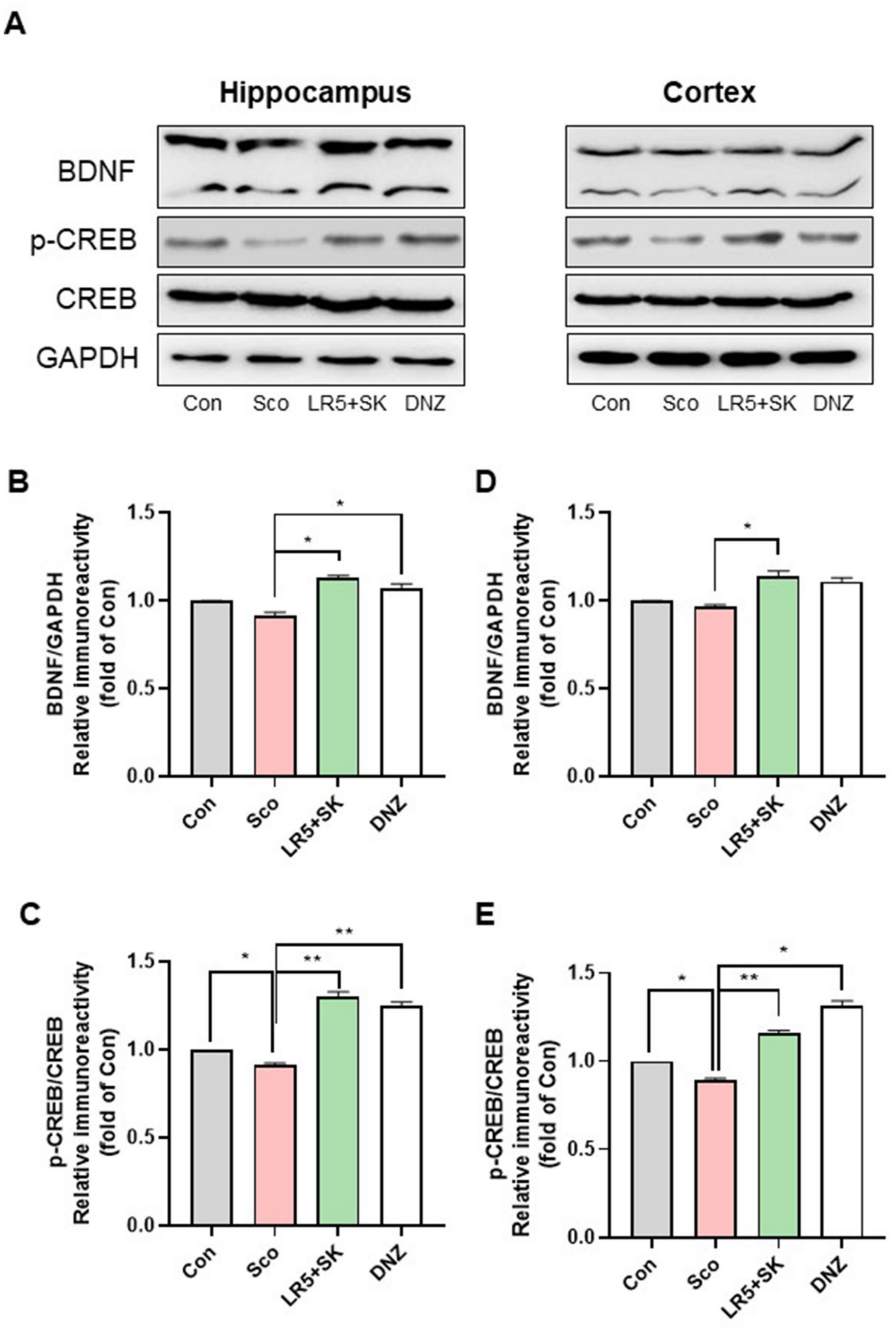
Effect of *L. rhamnosus* combined with skim milk on CREB-BDNF signaling pathway activation in hippocampal and cortical tissues. Representative Western blot images of phosphorylated CREB and BDNF **(A)** are presented. The protein expression levels of phosphorylated CREB **(B)** and BDNF **(C)**, in the hippocampus are presented. The protein expression levels of phosphorylated CREB **(D)** and BDNF **(E)**, in the cortex are presented. Statistical analysis was performed using one-way ANOVA followed by Dunnett’s T3 multiple comparisons test. The data represent the means ± S.E.M. (*n* = 4–5/group) (^*^p < 0.05, ^**^p < 0.01, ^***^p < 0.001; A: versus the other groups). Con, control; Sco, scopolamine; SK, skim milk; LR5, *L. rhamnosus* CBT LR5; DNZ, donepezil.

To elucidate the molecular mechanisms underlying the cognitive improvements induced by LR5 + SK treatment, we evaluated the mRNA and protein levels of BDNF, CREB, and TrkB in the hippocampal and cortical tissues (Figures 6, 7).

In the hippocampal tissues, qPCR analysis revealed significant group differences for CREB (*F*_3,28_ = 4.28, *p* < 0.05; Figure 6A), BDNF (*F*_3,28_ = 6.02, *p* < 0.01; Figure 6B) and TrkB (*F*_3,28_ = 2.226, *p* < 0.05; Figure 6C). Compared with the scopolamine group, the LR5 + SK group presented significantly restored expression levels of CREB, BDNF, and TrkB. We obtained similar findings in cortical tissues for CREB (*F*_3,28_ = 6.096, *p* < 0.01; Figure 6D), BDNF (*F*_3,28_ = 10.69, *p* < 0.001; Figure 6E) and TrkB (*F*_3,28_ = 8.368, *p* < 0.001; Figure 6F), with LR5 + SK treatment significantly increasing their expression.

Western blot analysis further confirmed these results, revealing significant increases in the phosphorylation of CREB (p-CREB, *F*_3,8_ = 121.3, *p* < 0.001, Figure 7B) and BDNF (*F*_3,8_ = 26.80, *p* < 0.001, Figure 7C) proteins in the hippocampus and cortex (CREB, one-way ANOVA, *F*_3,8_ = 119.6, *p* < 0.001, Figure 7D; BDNF, one-way ANOVA, *F*_3,8_ = 15.98, *p* < 0.001, Figure 7E) following LR5 + SK treatment. These findings indicate that LR5 combined with skim milk activates the CREB-BDNF signaling pathway in the brain, suggesting that this mechanism contributes significantly to its cognitive-enhancing effects under scopolamine-induced hypocholinergic conditions.

### 3.7 Correlation analysis between dominant fecal microbiota and cognitive deficit-related factors

**Figure 8:**
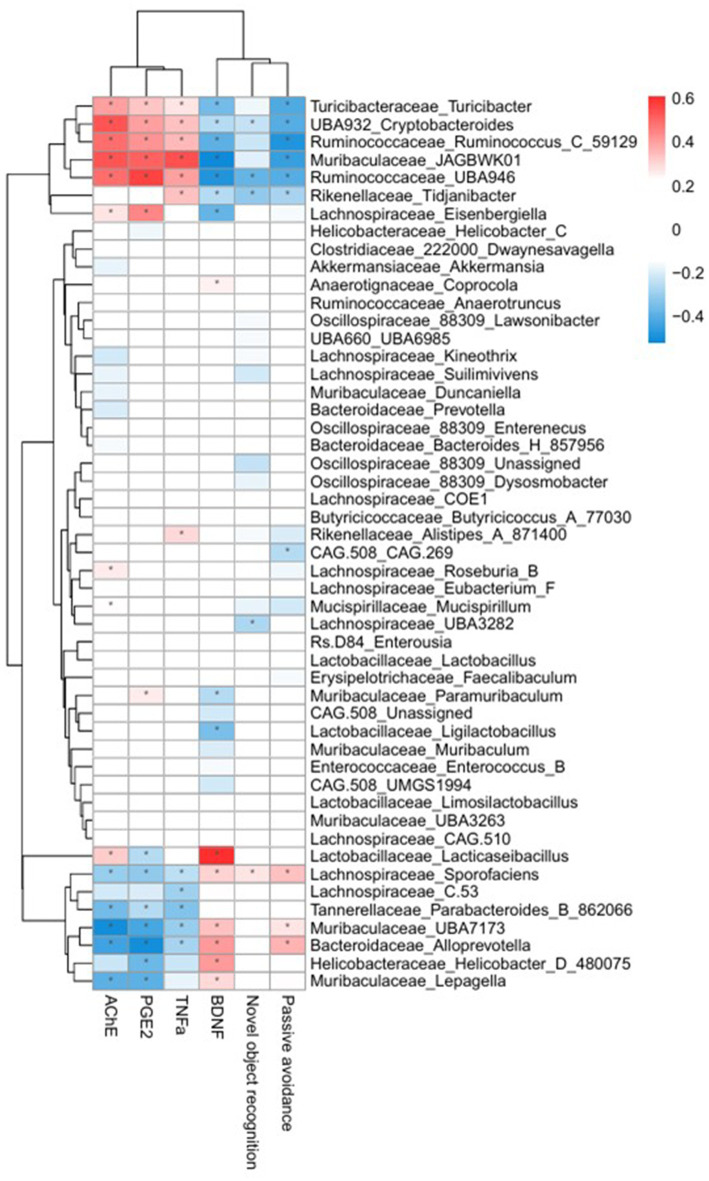
Heatmap illustrating correlations between the fecal microbiota and cognitive performance or inflammatory biomarkers. Red indicates positive correlations, and blue indicates negative correlations. Significant correlations (^*^p < 0.05, ^**^p < 0.01) are shown, highlighting associations between gut microbiota genera, cognitive performance indices (NOR discrimination ratio, PAT latency time), and serum biomarkers (AChE, PGE_2_, TNF-α, and BDNF). Con, control; Sco, scopolamine; SK, skim milk; LR5, *L. rhamnosus* CBT LR5; DNZ, donepezil.

To further elucidate potential gut microbiota and brain interactions, correlations between the dominant fecal microbiota and cognitive factors were analyzed and visualized as a heatmap (Figure 8). The relative abundance of the genus *Lacticaseibacillus* was significantly and positively correlated with the serum BDNF level. Conversely, the genera including *Turicibacter, Cryptobacteroides, Ruminococcus_C_59129, JAGBWK01* and *Tidjanibacter* presented significant negative correlations with cognitive performance metrics, including the discrimination ratio from the NOR test, latency from the PAT, and BDNF expression. Additionally, these genera were positively correlated with systemic inflammatory markers, such as PGE_2_ and TNF-*α*. These results suggest that specific gut microbiota genera are strongly associated with cognitive performance and systemic inflammation, supporting the hypothesis that microbiota modulation via LR5 combined with skim milk would be mechanistically involved in alleviating scopolamine-induced cognitive deficits.

## 4 Discussion

Numerous studies have demonstrated associations between probiotic or milk consumption and improved central nervous system (CNS) functions, including cognitive performance. However, the combined cognitive effects of probiotics with milk remain relatively unexplored. In the present study, we evaluated the effects of LR5 + SK treatment on cognitive performance and gut health in a scopolamine-induced animal model of AD. Our findings revealed that the administration of LR5, especially when combined with skim milk, effectively ameliorated scopolamine-induced cognitive deficits in both the NOR and the PAT. This improvement involves the modulation of the gut microbiota composition, the restoration of intestinal and BBB integrity through tight junction proteins, the attenuation of systemic inflammation, and the enhancement of BDNF-related signaling pathways.

Scopolamine is widely utilized to induce cognitive impairment in animal models due to its ability to cross the BBB and competitively inhibit muscarinic acetylcholine (ACh) receptors (Shim et al., 2022). This action leads to increased acetylcholinesterase (AChE) activity and reduces cholinergic neurotransmission, resulting in cognitive deficits that resemble the pathological features of AD (Chen et al., 2022; Zhang J. et al., 2024). Moreover, scopolamine acts as a vagal blocking agent, disrupting the vagus nerve, which is a critical anatomical structure that mediates communication between the gut and the brain, and is involved in regulating gut immunity, inflammation through the cholinergic anti-inflammatory pathway, and intestinal barrier function (Bonaz et al., 2019; Bonaz et al., 2018). Furthermore, scopolamine administration results in decreased expression levels of critical cognitive-related signaling molecules, such as BDNF and CREB (Yu et al., 2025; Qi et al., 2025). Notably, the peripheral reductions in BDNF levels correlate with neuroinflammation and impaired neuronal function (Lima Giacobbo et al., 2019; Ibrahim et al., 2022). Our results align with those of previous reports, demonstrating that chronic scopolamine administration significantly induced gut microbiota dysbiosis, disrupted intestinal barrier function, increased systemic inflammation, and decreased serum and brain BDNF levels, thereby validating the utility of this animal model for investigating probiotics and milk-based interventions for cognitive deficits.

LR5 has garnered substantial attention due to its diverse health-promoting effects, including the modulation of gastrointestinal function, metabolic improvements, immune regulation, and neuropsychological benefits (Shin et al., 2016; Kim et al., 2015). Previous clinical studies have demonstrated cognitive enhancements, including improvements in Alzheimer’s Disease Assessment Scale-Cognitive 13 (ADAS-Cog13) and Montreal Cognitive Assessment-Korea (MoCA-K) scores, and reduced plasma amyloid-beta (Aβ1-40/42) levels following LR5 supplementation combined with milk (Jung et al., 2025). Moreover, altered plasma Aβ40/Aβ42 ratios and tau phosphorylation levels are critical biomarkers associated with AD progression, with elevated ratios correlating positively with increased cognitive impairment severity (Scholl et al., 2024; Lee et al., 2019; Janelidze et al., 2016). Consistent with these reports, our study revealed that, compared with LR5 alone, LR5 combined with skim milk significantly improved recognition memory in the NOR and enhanced contextual long-term memory in the PAT, suggesting synergistic cognitive enhancement. Our results are consistent with emerging evidence that dietary prebiotic-probiotic combinations exert synergistic effects on gut-brain axis function. Skim milk, which contains lactose and oligosaccharides, serves as a natural probiotic substrate that enhances the survival and metabolic activity of probiotics such as LR5. In this context, recent work with *Lactiplantibacillus plantarum* MWFLp-182 demonstrated significant improvements in cognitive ability in a *D*-galactose-induced aging model, mediated by restoration of gut microbiota diversity and activation of the CREB-BDNF signaling pathway (Nie et al., 2024). These parallels suggest that the observed cognitive improvements with co-treatment of LR5 and skim milk may reflect a conserved mechanism whereby probiotic substrates potentiate probiotic efficacy to support neurocognitive health.

To elucidate the potential mechanisms underlying cognitive improvement, the diversity and composition of the gut microbiota were examined. Consistent with recent studies, we confirmed that scopolamine administration significantly reduced microbiota diversity (Zhang L. et al., 2024; Zheng et al., 2023; Ji et al., 2021). Importantly, LR5 treatment effectively restored microbiota diversity, an effect that was not observed with treatment with donepezil, a commonly used cholinesterase inhibitor in patients with AD (Li Y. et al., 2023; Jo et al., 2022). Specifically, scopolamine-induced reductions in beneficial bacterial genera, such as *Muribaculaceae_JAGBWK01* and *Muribaculaceae_Duncaniella*, were significantly reversed by LR5 supplementation, particularly when combined with skim milk. Notably, *Muribaculaceae_UBA3263* is a beneficial genus associated with improved gut barrier integrity, metabolite production, anti-inflammatory, antioxidant and antistress properties and longevity (Shenghua et al., 2020; Ormerod et al., 2016). Whereas, a decrease in *Muribaculaceae* abundance has been linked to several diseases. Clinical studies have demonstrated that the abundance of *Muribaculaceae* is reduced in various inflammatory diseases, including IBD and type 1 diabetes (Krych et al., 2015; Rooks et al., 2014). These microbiota alterations likely contribute to improved intestinal barrier function and reduced inflammation, thereby supporting cognitive function.

Correlation analysis further revealed an association between microbiota composition and cognitive biomarkers. The abundance of *Lacticaseibacillus* was positively correlated with the serum BDNF level, suggesting direct or indirect beneficial effects on neuronal health. In contrast, genera such as *Turicibacter*, *Cryptobacteroides*, *Ruminococcus_C_59129*, *Muribaculaceae_JAGBWK01*, and *Tidjanibacter* were negatively correlated with cognitive performance metrics, including the discrimination ratio from NOR, latency from PAT and BDNF expression, whereas they were positively correlated with inflammatory biomarkers, such as AChE, PGE_2_ and TNF-*α* levels in serum. Previous clinical and preclinical studies have reported elevated levels of *Turicibacter* in individuals with autism spectrum disorder (Gerges et al., 2024) and AD (Vogt et al., 2017), while decreased levels of *Ruminococcus* are associated with amyloid-positive mild cognitive impairment (MCI) compared with healthy controls (Hung et al., 2023). The observed increase in BDNF expression following administration of LR5 combined with skim milk is likely mediated through multiple, converging mechanisms. Restoration of gut microbiota composition, including enrichment of beneficial genera such as *Muribaculaceae* and *Lacticaseibacillus*, may directly promote neurotrophic signaling via microbial metabolites and vagal pathways. At the same time, attenuation of systemic inflammation (reduced TNF-α and PGE₂) and oxidative stress likely provides an indirect but complementary route for preserving neuronal health and facilitating CREB–BDNF–TrkB activation. In particular, oxidative stress is a critical driver of neurodegeneration, as excess ROS production exacerbates synaptic dysfunction and accelerates neuronal loss (Wen et al., 2025). Probiotic interventions have been shown to mitigate oxidative damage by enhancing endogenous antioxidant defenses and modulating gut-derived metabolites. In line with this, recent work on *Lactiplantibacillus plantarum* MWFLp-182 demonstrated protective effects against oxidative deficits induced by 2,2′-Azobis (2-methylpropionamidine) dihydrochloride, accompanied by regulation of key antioxidant-related genes (Nie et al., 2025). These findings suggest that the cognitive improvements observed with LR5 + skim milk may also involve attenuation of oxidative stress, acting synergistically with the restoration of gut microbiota balance and reduction of systemic inflammation.

Notably, our results revealed significant improvements in gut barrier integrity upon LR5 supplementation, particularly in combination with skim milk, as demonstrated by elevated expression of tight junction proteins, including claudin-1, occludin and ZO-1, in ileum and hippocampal tissues. These effects corresponded with decreased systemic inflammation, as evidenced by reduced serum levels of PGE_2_ and TNF-α. Simultaneously, enhanced BBB integrity may reduce neuroinflammation and neuronal damage, ultimately improving cognitive outcomes (Hung et al., 2023; Zhang et al., 2023). Furthermore, molecular analysis of hippocampal and cortical tissues revealed that LR5 combined with skim milk significantly enhanced the CREB- BDNF–TrkB signaling pathway, which is essential for synaptic plasticity, neurogenesis, and cognitive function (Li et al., 2022; Amidfar et al., 2020). These data align with previous reports highlighting the importance of CREB-BDNF signaling in neuroprotection and cognitive resilience under conditions of neurodegeneration and cholinergic dysfunction. Our findings that LR5 combined with skim milk significantly activated CREB–BDNF–TrkB signaling in hippocampal and cortical tissues are consistent with previous reports highlighting the neuroprotective roles of probiotics in cognitive decline. Recent studies have demonstrated that probiotic supplementation enhances neurogenesis and synaptic plasticity via upregulation of CREB–BDNF pathways, thereby improving memory performance in neurodegenerative and aging models (Olajide and Ijomone, 2025; Prajapati et al., 2025). These data reinforce the notion that probiotics can serve as modulators of neurotrophic signaling and cognitive resilience.

## 5 Conclusion

Collectively, our findings provide comprehensive mechanistic insights supporting the efficacy of combined LR5 and milk supplementation in ameliorating cognitive impairment through the modulation of the gut microbiome, enhancement of barrier integrity, attenuation of systemic inflammation, and stimulation of neurotrophic signaling. Therefore, the combination of LR5 with milk represents a promising therapeutic strategy to improve cognitive function in patients with AD and potentially other neurodegenerative conditions characterized by gut-brain axis dysregulation.

## Data Availability

The datasets presented in this study can be found in online repositories. The statistical anaylsis of the data can be found in the article/Supplementary Table S1.

## References

[ref1] Akhgarjand C. Vahabi Z. Shab-Bidar S. Etesam F. Djafarian K. (2022). Effects of probiotic supplements on cognition, anxiety, and physical activity in subjects with mild and moderate Alzheimer's disease: a randomized, double-blind, and placebo-controlled study. Front. Aging Neurosci. 14:1032494. doi: 10.3389/fnagi.2022.1032494, PMID: 36389063 PMC9647197

[ref2] Aljumaah M. R. Bhatia U. Roach J. Gunstad J. Azcarate Peril M. A. (2022). The gut microbiome, mild cognitive impairment, and probiotics: a randomized clinical trial in middle-aged and older adults. Clin. Nutr. 41, 2565–2576. doi: 10.1016/j.clnu.2022.09.012, PMID: 36228569

[ref3] Amidfar M. de Oliveira J. Kucharska E. Budni J. Kim Y. K. (2020). The role of CREB and BDNF in neurobiology and treatment of Alzheimer's disease. Life Sci. 257:118020. doi: 10.1016/j.lfs.2020.118020, PMID: 32603820

[ref4] Ansari A. Son D. Hur Y. M. Park S. You Y. A. Kim S. M.. (2023). *Lactobacillus* probiotics improve vaginal Dysbiosis in asymptomatic women. Nutrients 15:1862. doi: 10.3390/nu15081862, PMID: 37111086 PMC10143682

[ref5] Bae H. J. Kim J. Y. Choi S. H. Kim S. Y. Kim H. J. Cho Y. E.. (2023). Paeonol, the active component of *Cynanchum paniculatum*, ameliorated schizophrenia-like behaviors by regulating the PI3K-Akt-GSK3β-NF-κB signalling pathway in MK-801-treated mice. J. Ethnopharmacol. 314:116627. doi: 10.1016/j.jep.2023.116627, PMID: 37164258

[ref6] Bae H. J. Kim J. Kim J. Goo N. Cai M. Cho K.. (2020). The effect of maslinic acid on cognitive dysfunction induced by cholinergic blockade in mice. Br. J. Pharmacol. 177, 3197–3209. doi: 10.1111/bph.15042, PMID: 32133639 PMC7312314

[ref7] Barbara G. Barbaro M. R. Fuschi D. Palombo M. Falangone F. Cremon C.. (2021). Inflammatory and microbiota-related regulation of the intestinal epithelial barrier. Front. Nutr. 8:718356. doi: 10.3389/fnut.2021.718356, PMID: 34589512 PMC8475765

[ref8] Bhat M. I. Sowmya K. Kapila S. Kapila R. (2020). Potential probiotic *Lactobacillus rhamnosus* (MTCC-5897) inhibits *Escherichia coli* impaired intestinal barrier function by modulating the host tight junction gene response. Probiotics Antimicrob. Proteins 12, 1149–1160. doi: 10.1007/s12602-019-09608-8, PMID: 31732863

[ref9] Bonaz B. Bazin T. Pellissier S. (2018). The vagus nerve at the interface of the microbiota-gut-brain axis. Front. Neurosci. 12:49. doi: 10.3389/fnins.2018.00049, PMID: 29467611 PMC5808284

[ref10] Bonaz B. Sinniger V. Pellissier S. (2019). Vagus nerve stimulation at the interface of brain-gut interactions. Cold Spring Harb. Perspect. Med. 9:a034199. doi: 10.1101/cshperspect.a034199, PMID: 30201788 PMC6671930

[ref11] Chelakkot C. Ghim J. Ryu S. H. (2018). Mechanisms regulating intestinal barrier integrity and its pathological implications. Exp. Mol. Med. 50, 1–9. doi: 10.1038/s12276-018-0126-x, PMID: 30115904 PMC6095905

[ref12] Chen Z. R. Huang J. B. Yang S. L. Hong F. F. (2022). Role of cholinergic signaling in Alzheimer's disease. Molecules 27:1816. doi: 10.3390/molecules27061816, PMID: 35335180 PMC8949236

[ref13] Demir R. Saritas S. Bechelany M. Karav S. (2025). Lactoferrin: properties and potential uses in the food industry. Int. J. Mol. Sci. 26:1404. doi: 10.3390/ijms26041404 40003872 PMC11855648

[ref14] Dong Y. Fan H. Zhang Z. Jiang F. Li M. Zhou H.. (2022). Berberine ameliorates DSS-induced intestinal mucosal barrier dysfunction through microbiota-dependence and Wnt/beta-catenin pathway. Int. J. Biol. Sci. 18, 1381–1397. doi: 10.7150/ijbs.65476 35280677 PMC8898376

[ref15] Feng J. Cen Q. Cui Y. Hu X. Li M. Wang L.. (2025). *Lactobacillus rhamnosus*: An emerging probiotic with therapeutic potential for depression. Pharmacol. Res. 211:107541. doi: 10.1016/j.phrs.2024.107541, PMID: 39653301

[ref16] Gallo V. Arienzo A. Tomassetti F. Antonini G. (2024). Milk bioactive compounds and gut microbiota modulation: the role of whey proteins and milk oligosaccharides. Foods 13:907. doi: 10.3390/foods13060907, PMID: 38540897 PMC10969594

[ref17] Gerges P. Bangarusamy D. K. Bitar T. Alameddine A. Nemer G. Hleihel W. (2024). *Turicibacter* and *Catenibacterium* as potential biomarkers in autism spectrum disorders. Sci. Rep. 14:23184. doi: 10.1038/s41598-024-73700-5, PMID: 39369020 PMC11455930

[ref18] Ghosh S. Whitley C. S. Haribabu B. Jala V. R. (2021). Regulation of intestinal barrier function by microbial metabolites. Cell. Mol. Gastroenterol. Hepatol. 11, 1463–1482. doi: 10.1016/j.jcmgh.2021.02.007, PMID: 33610769 PMC8025057

[ref19] Guimaraes A. P. Uliana D. S. Sant'Ana M. R. de Sao Jose J. F. B. (2025). *Lacticaseibacillus rhamnosus*: an overview of the viability in fruit and vegetable juices and their potential effects on human health. Probiotics Antimicrob. Proteins 17, 1905–1920. doi: 10.1007/s12602-025-10454-0, PMID: 39904828

[ref20] Hung C. C. Chao Y. P. Lee Y. Huang C. W. Huang S. H. Chang C. C.. (2023). Cingulate white matter mediates the effects of fecal *Ruminococcus* on neuropsychiatric symptoms in patients with amyloid-positive amnestic mild cognitive impairment. BMC Geriatr. 23:720. doi: 10.1186/s12877-023-04417-9, PMID: 37936084 PMC10631051

[ref21] Ibrahim A. M. Chauhan L. Bhardwaj A. Sharma A. Fayaz F. Kumar B.. (2022). Brain-derived neurotropic factor in neurodegenerative disorders. Biomedicine 10:1143. doi: 10.3390/biomedicines10051143, PMID: 35625880 PMC9138678

[ref22] Isik M. Kose F. Ozbayer C. Budak O. Kaya R. K. Erdogan D. G.. (2025). Promising antidepressant potential: the role of *Lactobacillus rhamnosus* GG in mental health and stress response. Probiotics Antimicrob. Proteins. doi: 10.1007/s12602-025-10470-0 PMC12634810 39962033

[ref23] Janelidze S. Stomrud E. Palmqvist S. Zetterberg H. van Westen D. Jeromin A.. (2016). Plasma β-amyloid in Alzheimer's disease and vascular disease. Sci. Rep. 6:26801. doi: 10.1038/srep26801, PMID: 27241045 PMC4886210

[ref24] Ji J. Yi X. Zhu Y. Yu H. Huang S. Liu Z.. (2021). Tilapia head protein hydrolysate attenuates scopolamine-induced cognitive impairment through the gut-brain axis in mice. Foods 10:3129. doi: 10.3390/foods10123129, PMID: 34945680 PMC8701847

[ref25] Jo J. K. Lee G. Nguyen C. D. Park S. E. Kim E. J. Kim H. W.. (2022). Effects of donepezil treatment on brain metabolites, gut microbiota, and gut metabolites in an amyloid β-induced cognitive impairment mouse pilot model. Molecules 27:6591. doi: 10.3390/molecules27196591 36235127 PMC9572896

[ref26] Jung S. J. Cho K. Jung E. S. Son D. Byun J. S. Kim S. I.. (2025). Augmenting cognitive function in the elderly with mild cognitive impairment using probiotic *Lacticaseibacillus rhamnosus* CBT-LR5: a 12-week randomized, double-blind, parallel-group non-comparative study. Nutrients 17:691. doi: 10.3390/nu17040691, PMID: 40005019 PMC11858765

[ref27] Kim H. J. Park K. W. Kim T. E. Im J. Y. Shin H. S. Kim S.. (2015). Elevation of the plasma Aβ40/Aβ42 ratio as a diagnostic marker of sporadic early-onset Alzheimer's disease. J Alzheimer's Dis 48, 1043–1050. doi: 10.3233/JAD-143018, PMID: 26444752

[ref28] Krych L. Nielsen D. S. Hansen A. K. Hansen C. H. (2015). Gut microbial markers are associated with diabetes onset, regulatory imbalance, and IFN-γ level in NOD mice. Gut Microbes 6, 101–109. doi: 10.1080/19490976.2015.1011876, PMID: 25648687 PMC4615729

[ref29] Kurland D. B. Gerzanich V. Karimy J. K. Woo S. K. Vennekens R. Freichel M.. (2016). The Sur1-Trpm4 channel regulates NOS2 transcription in TLR4-activated microglia. J. Neuroinflammation 13:130. doi: 10.1186/s12974-016-0599-2, PMID: 27246103 PMC4888589

[ref30] Lee J. C. Kim S. J. Hong S. Kim Y. (2019). Diagnosis of Alzheimer's disease utilizing amyloid and tau as fluid biomarkers. Exp. Mol. Med. 51, 1–10. doi: 10.1038/s12276-019-0250-2, PMID: 31073121 PMC6509326

[ref31] Li Y. Li F. Qin D. Chen H. Wang J. Wang J.. (2022). The role of brain derived neurotrophic factor in central nervous system. Front. Aging Neurosci. 14:986443. doi: 10.3389/fnagi.2022.986443, PMID: 36158555 PMC9493475

[ref32] Li Y. Wu M. Kong M. Sui S. Wang Q. He Y.. (2023). Impact of donepezil supplementation on Alzheimer's disease-like pathology and gut microbiome in APP/PS1 mice. Microorganisms 11:2306. doi: 10.3390/microorganisms11092306, PMID: 37764150 PMC10537997

[ref33] Li X. Zheng P. Cao W. Cao Y. She X. Yang H.. (2023). *Lactobacillus rhamnosus* GG ameliorates noise-induced cognitive deficits and systemic inflammation in rats by modulating the gut-brain axis. Front. Cell. Infect. Microbiol. 13:1067367. doi: 10.3389/fcimb.2023.1067367, PMID: 37180445 PMC10169735

[ref34] Lima Giacobbo B. Doorduin J. Klein H. C. Dierckx R. Bromberg E. de Vries E. F. J. (2019). Brain-derived neurotrophic factor in brain disorders: focus on neuroinflammation. Mol. Neurobiol. 56, 3295–3312. doi: 10.1007/s12035-018-1283-6 30117106 PMC6476855

[ref35] Liu S. Gao J. Zhu M. Liu K. Zhang H. L. (2020). Gut microbiota and dysbiosis in Alzheimer's disease: implications for pathogenesis and treatment. Mol. Neurobiol. 57, 5026–5043. doi: 10.1007/s12035-020-02073-3, PMID: 32829453 PMC7541367

[ref36] Loh J. S. Mak W. Q. Tan L. K. S. Ng C. X. Chan H. H. Yeow S. H.. (2024). Microbiota-gut-brain axis and its therapeutic applications in neurodegenerative diseases. Signal Transduct. Target. Ther. 9:37. doi: 10.1038/s41392-024-01743-1, PMID: 38360862 PMC10869798

[ref37] Ni H. Liu M. Cao M. Zhang L. Zhao Y. Yi L.. (2024). Sinomenine regulates the cholinergic anti-inflammatory pathway to inhibit TLR4/NF-κB pathway and protect the homeostasis in brain and gut in scopolamine-induced Alzheimer's disease mice. Biomed. Pharmacother. 171:116190. doi: 10.1016/j.biopha.2024.116190, PMID: 38278026

[ref38] Nie H. Ma X. T. Kong F. Y. Luo Y. H. Mu G. Q. Wu X. M. (2025). Improvement of MWFLp-182 on oxidative deficits induced by in 2,2′-azobis(2-methylpropionamidine) dihydrochloride and the relating key gene analysis. Food Sci. Hum. Well. 14:9250026. doi: 10.26599/FSHW.2024.9250026

[ref39] Nie H. Wang X. Luo Y. Kong F. Mu G. Wu X. (2024). Mechanism explanation on improved cognitive ability of D-gal inducing aged mice model by *Lactiplantibacillus plantarum* MWFLp-182 via the microbiota-gut-brain Axis. J. Agric. Food Chem. 72, 9795–9806. doi: 10.1021/acs.jafc.3c09675, PMID: 38608178

[ref40] Olajide T. S. Ijomone O. M. (2025). Targeting gut microbiota as a therapeutic approach for neurodegenerative diseases. Neuroprotection 3, 120–130. doi: 10.1002/nep3.70000, PMID: 40589476 PMC12208687

[ref41] Ormerod K. L. Wood D. L. Lachner N. Gellatly S. L. Daly J. N. Parsons J. D.. (2016). Genomic characterization of the uncultured *Bacteroidales* family S24-7 inhabiting the guts of homeothermic animals. Microbiome 4:36. doi: 10.1186/s40168-016-0181-2, PMID: 27388460 PMC4936053

[ref42] Pang B. Zhu Z. Xiao C. Luo Y. Fang H. Bai Y.. (2022). Keratin 17 is required for lipid metabolism in keratinocytes and benefits epidermal permeability barrier homeostasis. Front. Cell Dev. Biol. 9:779257. doi: 10.3389/fcell.2021.779257, PMID: 35096815 PMC8790522

[ref43] Prajapati S. K. Wang S. Mishra S. P. Jain S. Yadav H. (2025). Protection of Alzheimer's disease progression by a human-origin probiotics cocktail. Sci. Rep. 15:1589. doi: 10.1038/s41598-024-84780-8, PMID: 39794404 PMC11724051

[ref44] Qi Y. Wang Y. Ni M. He Y. Li L. Hu Y. (2025). Safflower yellow alleviates cognitive impairment in mice by modulating cholinergic system function, oxidative stress, and CREB/BDNF/TrkB signaling pathway. J. Ethnopharmacol. 340:118986. doi: 10.1016/j.jep.2024.118986, PMID: 39461389

[ref45] Rob M. Yousef M. Lakshmanan A. P. Mahboob A. Terranegra A. Chaari A. (2025). Microbial signatures and therapeutic strategies in neurodegenerative diseases. Biomed. Pharmacother. 184:117905. doi: 10.1016/j.biopha.2025.117905, PMID: 39933444

[ref46] Rooks M. G. Veiga P. Wardwell-Scott L. H. Tickle T. Segata N. Michaud M.. (2014). Gut microbiome composition and function in experimental colitis during active disease and treatment-induced remission. ISME J. 8, 1403–1417. doi: 10.1038/ismej.2014.3, PMID: 24500617 PMC4069400

[ref47] Scholl M. Verberk I. M. W. Del Campo M. Delaby C. Therriault J. Chong J. R.. (2024). Challenges in the practical implementation of blood biomarkers for Alzheimer's disease. Lancet Healthy Longev. 5:100630. doi: 10.1016/j.lanhl.2024.07.013, PMID: 39369727

[ref48] Schoultz I. Keita A. V. (2020). The intestinal barrier and current techniques for the assessment of gut permeability. Cells 9:1909. doi: 10.3390/cells9081909, PMID: 32824536 PMC7463717

[ref49] Shenghua P. Ziqin Z. Shuyu T. Huixia Z. Xianglu R. Jiao G. (2020). An integrated fecal microbiome and metabolome in the aged mice reveal anti-aging effects from the intestines and biochemical mechanism of FuFang zhenshu TiaoZhi (FTZ). Biomed. Pharmacother. 121:109421. doi: 10.1016/j.biopha.2019.109421, PMID: 31743877

[ref50] Shim K. H. Kang M. J. Sharma N. An S. S. A. (2022). Beauty of the beast: anticholinergic tropane alkaloids in therapeutics. Nat. Prod. Bioprospect. 12:33. doi: 10.1007/s13659-022-00357-w, PMID: 36109439 PMC9478010

[ref51] Shin H. S. Lee S. K. Kim S. Kim H. J. Chae W. S. Park S. A. (2016). The correlation study between plasma Abeta proteins and cerebrospinal fluid Alzheimer's disease biomarkers. Dement. Neurocogn. Disord. 15, 122–128. doi: 10.12779/dnd.2016.15.4.122 30906353 PMC6428019

[ref52] Tette F. M. Kwofie S. K. Wilson M. D. (2022). Therapeutic anti-depressant potential of microbial GABA produced by *Lactobacillus rhamnosus* strains for GABAergic signaling restoration and inhibition of addiction-induced HPA axis hyperactivity. Curr. Issues Mol. Biol. 44, 1434–1451. doi: 10.3390/cimb44040096, PMID: 35723354 PMC9164062

[ref53] Turabelidze A. Guo S. DiPietro L. A. (2010). Importance of housekeeping gene selection for accurate reverse transcription-quantitative polymerase chain reaction in a wound healing model. Wound Repair Regen. 18, 460–466. doi: 10.1111/j.1524-475X.2010.00611.x, PMID: 20731795 PMC2939911

[ref54] Vogt N. M. Kerby R. L. Dill-McFarland K. A. Harding S. J. Merluzzi A. P. Johnson S. C.. (2017). Gut microbiome alterations in Alzheimer's disease. Sci. Rep. 7:13537. doi: 10.1038/s41598-017-13601-y, PMID: 29051531 PMC5648830

[ref55] Walsh C. Lane J. A. van Sinderen D. Hickey R. M. (2020). Human milk oligosaccharides: shaping the infant gut microbiota and supporting health. J. Funct. Foods 72:104074. doi: 10.1016/j.jff.2020.104074, PMID: 32834834 PMC7332462

[ref56] Wang L. Zhao R. Li X. Shao P. Xie J. Su X.. (2024). *Lactobacillus rhamnosus* GG improves cognitive impairments in mice with sepsis. PeerJ 12:e17427. doi: 10.7717/peerj.17427, PMID: 38827289 PMC11141560

[ref57] Wen P. Sun Z. Gou F. Wang J. Fan Q. Zhao D.. (2025). Oxidative stress and mitochondrial impairment: key drivers in neurodegenerative disorders. Ageing Res. Rev. 104:102667. doi: 10.1016/j.arr.2025.102667, PMID: 39848408

[ref58] Xiao-Hang Q. Si-Yue C. Hui-Dong T. (2024). Multi-strain probiotics ameliorate Alzheimer's-like cognitive impairment and pathological changes through the AKT/GSK-3β pathway in senescence-accelerated mouse prone 8 mice. Brain Behav. Immun. 119, 14–27. doi: 10.1016/j.bbi.2024.03.031, PMID: 38548184

[ref59] Xie J. Van Hoecke L. Vandenbroucke R. E. (2021). The impact of systemic inflammation on Alzheimer's disease pathology. Front. Immunol. 12:796867. doi: 10.3389/fimmu.2021.796867, PMID: 35069578 PMC8770958

[ref60] Yu Y. J. Rahman M. U. Balakrishnan R. Kim J. M. Kim J. H. Choi D. K. (2025). The novel peptide DBCH reduces LPS-stimulated NF-κB/MAPK signaling in BV-2 microglia and ameliorates cognitive impairment in scopolamine-treated mice by modulating BDNF/CREB. Neurochem. Int. 185:105946. doi: 10.1016/j.neuint.2025.105946, PMID: 39971241

[ref61] Zhang L. Jiang Z. Hu S. Ni H. Zhao Y. Tan X.. (2024). GSK3beta substrate-competitive inhibitors regulate the gut homeostasis and barrier function to inhibit neuroinflammation in scopolamine-induced Alzheimer's disease model mice. Inflammation 48, 1438–1459. doi: 10.1007/s10753-024-02133-z 39180577

[ref62] Zhang W. Xiao D. Mao Q. Xia H. (2023). Role of neuroinflammation in neurodegeneration development. Signal Transduct. Target. Ther. 8:267. doi: 10.1038/s41392-023-01486-5, PMID: 37433768 PMC10336149

[ref63] Zhang J. Zhang Y. Wang J. Xia Y. Zhang J. Chen L. (2024). Recent advances in Alzheimer's disease: mechanisms, clinical trials and new drug development strategies. Signal Transduct. Target. Ther. 9:211. doi: 10.1038/s41392-024-01911-3, PMID: 39174535 PMC11344989

[ref64] Zheng J. Ahmad A. A. Yang Y. Liang Z. Shen W. Feng M.. (2022). *Lactobacillus rhamnosus* CY12 enhances intestinal barrier function by regulating tight junction protein expression, oxidative stress, and inflammation response in lipopolysaccharide-induced Caco-2 cells. Int. J. Mol. Sci. 23:11162. doi: 10.3390/ijms231911162, PMID: 36232464 PMC9569798

[ref65] Zheng J. Y. Kang T. Jiang C. Lin L. K. Gao L. Jin L. H.. (2023). Gut microbiome and brain transcriptome analyses reveal the effect of walnut oil in preventing scopolamine-induced cognitive impairment. Food Funct. 14, 9707–9724. doi: 10.1039/D3FO01893H, PMID: 37814808

[ref66] Zheng Y. Qin C. Wen M. Zhang L. Wang W. (2024). The effects of food nutrients and bioactive compounds on the gut microbiota: a comprehensive review. Foods 13:1345. doi: 10.3390/foods13091345, PMID: 38731716 PMC11083588

