# Supplementary material for: Lacticaseibacillus rhamnosus CBT LR5 with skim milk alleviates scopolamine-induced cognitive impairment in mice
Source: Front Microbiol. 2025 Oct 17;16:1672153. doi: 10.3389/fmicb.2025.1672153 (PMC12575242; doi:10.3389/fmicb.2025.1672153)
Supplement: Supplementary file 1 [file Datasheet_1.docx]

**Supplementary methods**

Open filed test (OFT)

Black square Plexiglas boxes (w × d × h: 41.5 × 41.5 × 41.5 cm) equipped with the video-based Ethovision system (Noldus, Wageningen, The Netherlands) were used in the open field task. MK-801 (0.2 mg/kg, i.p.) or aripiprazole (1 mg/kg, i.p.) was administered 30 min before the behavioral tasks. HCE (30, 100 or 300 mg/kg, p.o.) or the vehicle was administered 1 h before each task. Mice were moved to the middle of the box and allowed to explore for 30 min. The spontaneous locomotor behaviors were recorded in order to evaluate the horizontal locomotor activity. The apparatus was cleaned with 70% ethanol after each trial. The locomotor activity was expressed as the total ambulatory distance.

**Supplementary Fig. S1**

During the treatment, the body weights for all the experimental animals were measured every 5 days. The body weight showed that there were no significant differences between the treatment groups. These results suggesting that LR5 and the other materials has no adverse reactions or toxicity. Statistical analysis was performed using one-way ANOVA followed by Turkey’s multiple comparisons test. The data represent the means ± S.E.M.s (n = 10 per group). Con, control. Sco, scopolamine. SK, skim milk. LR5, *L. rhamnosus* CBT LR5. DNZ, donepezil.

**Supplementary Fig. S2**

**A**

**B**

(A) Distance moved (cm) measured at 5 min intervals over a 30 min period. (B) Total distance moved (cm) during the entire 30 min testing session. No significant differences were observed among the treatment groups. Statistical analysis was performed using one-way ANOVA followed by Turkey’s multiple comparisons test. The data represent the means ± S.E.M.s (n = 10 per group). Con, control. Sco, scopolamine. SK, skim milk. LR5, *L. rhamnosus* CBT LR5. DNZ, donepezil. N.S., no significant.

**Supplementary Fig. S3**

**A**

**
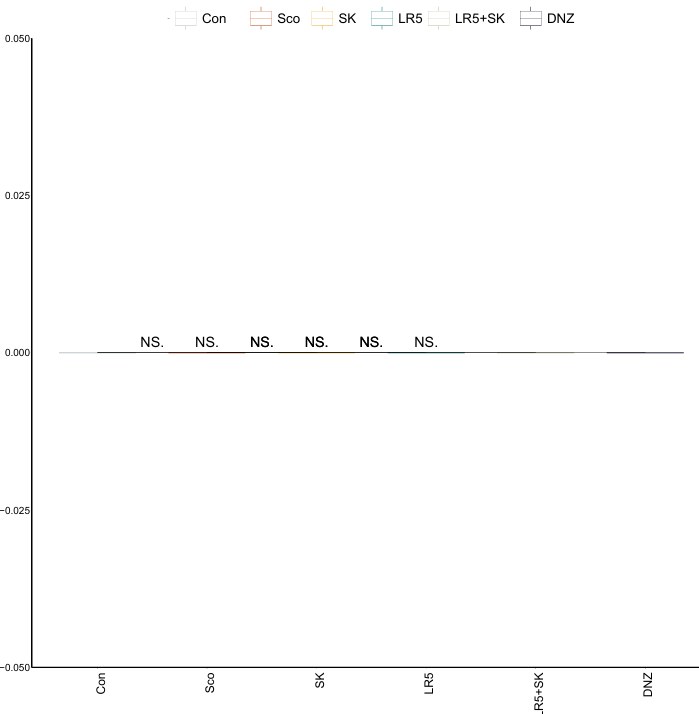
**

**B**

**
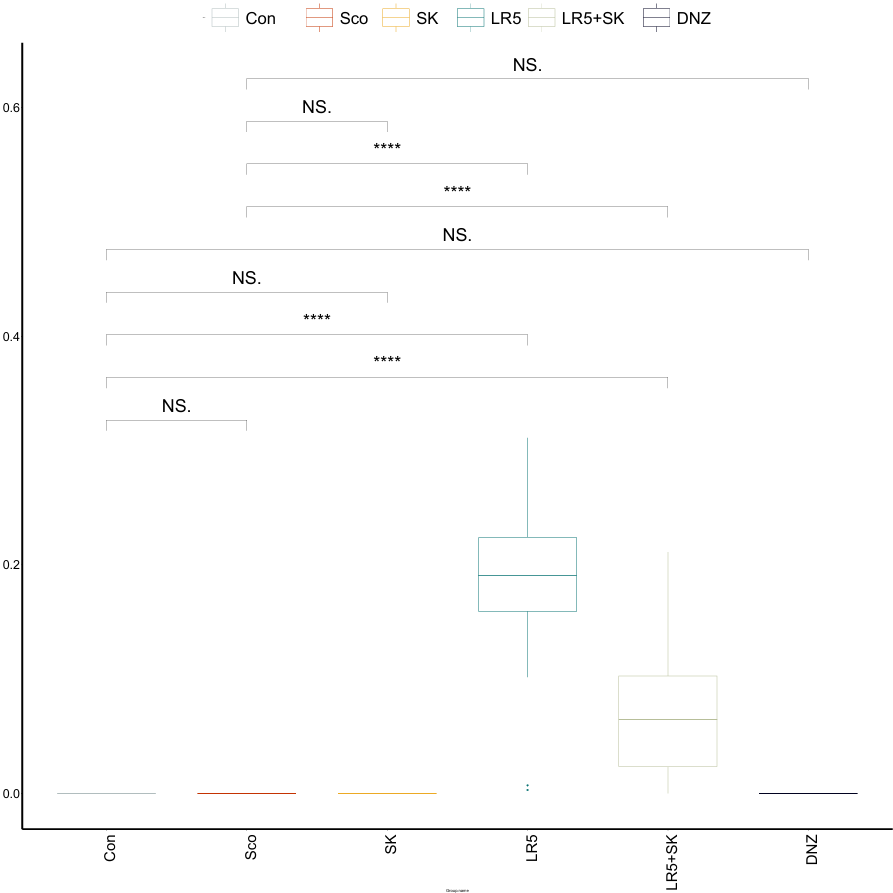
**

**C**

**
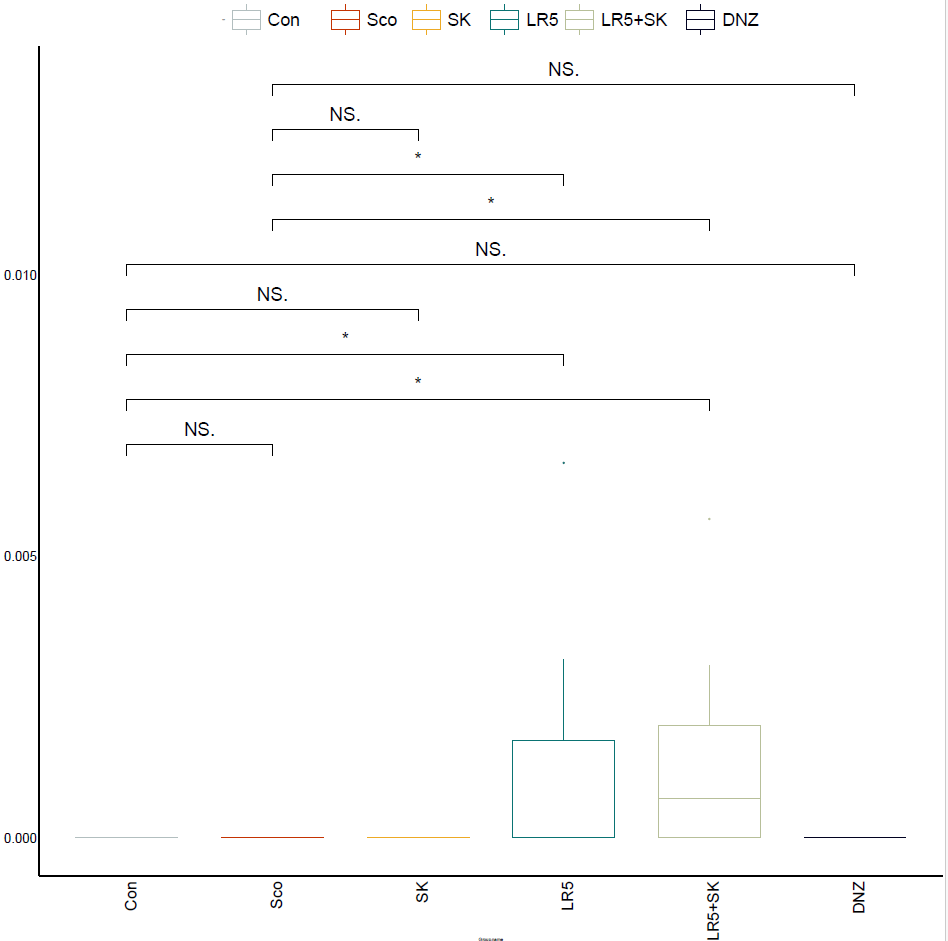
**

The relative abundance of the genus *Lacticaseibacillus* in fecal samples based on the 16s rRNA sequencing. The levels of *Lacticaseibacillus* in fecal sample collected at (A) 0 week, (B) 4 week, and (C) 6 week are presented. Statistical analysis was performed using one-way ANOVA followed by Dunnett’s T3 multiple comparisons test. The data represent the means ± S.E.M. (n = 9–10/group) (*P < 0.05, **P < 0.01, ***P < 0.001; A: versus between each group). Con, control. Sco, scopolamine. SK, skim milk. LR5, *L. rhamnosus* CBT LR5. DNZ, donepezil. N.S., no significant.
